# Can antibiotic prescriptions in respiratory tract infections be improved? A cluster-randomized educational intervention in general practice – The Prescription Peer Academic Detailing (Rx-PAD) Study [NCT00272155]

**DOI:** 10.1186/1472-6963-6-75

**Published:** 2006-06-15

**Authors:** Svein Gjelstad, Arne Fetveit, Jørund Straand, Ingvild Dalen, Sture Rognstad, Morten Lindbaek

**Affiliations:** 1 Department of General Practice and Community Medicine, University of Oslo, PO Box 1130 Blindern, 0317 Oslo, Norway; 2 Institute of Basic Medical Sciences, Department of Biostatistics, University of Oslo, PO Box 1122 Blindern, 0317 Oslo, Norway

## Abstract

**Background:**

More than half of all antibiotic prescriptions in general practice are issued for respiratory tract infections (RTIs), despite convincing evidence that many of these infections are caused by viruses. Frequent misuse of antimicrobial agents is of great global health concern, as we face an emerging worldwide threat of bacterial antibiotic resistance. There is an increasing need to identify determinants and patterns of antibiotic prescribing, in order to identify where clinical practice can be improved.

**Methods/Design:**

Approximately 80 peer continuing medical education (CME) groups in southern Norway will be recruited to a cluster randomized trial. Participating groups will be randomized either to an intervention- or a control group. A multifaceted intervention has been tailored, where key components are educational outreach visits to the CME-groups, work-shops, audit and feedback. Prescription Peer Academic Detailers (Rx-PADs), who are trained GPs, will conduct the educational outreach visits. During these visits, evidence-based recommendations of antibiotic prescriptions for RTIs will be presented and software will be handed out for installation in participants PCs, enabling collection of prescription data. These data will subsequently be linked to corresponding data from the Norwegian Prescription Database (NorPD). Individual feedback reports will be sent all participating GPs during and one year after the intervention. Main outcomes are baseline proportion of inappropriate antibiotic prescriptions for RTIs and change in prescription patterns compared to baseline one year after the initiation of the tailored pedagogic intervention.

**Discussion:**

Improvement of prescription patterns in medical practice is a challenging task. A thorough evaluation of guidelines for antibiotic treatment in RTIs may impose important benefits, whereas inappropriate prescribing entails substantial costs, as well as undesirable consequences like development of antibiotic resistance. Our hypothesis is that an educational intervention program will be effective in improving prescription patterns by reducing the total number of antibiotic prescriptions, as well as reducing the amount of broad-spectrum antibiotics, with special emphasis on macrolides.

## Background

Acute respiratory tract infections (RTIs) are important targets for educational strategies aimed at reducing inappropriate prescriptions of antibiotics, because such infections have a high prevalence in general practice [1], and because antibiotics are commonly prescribed even for those illnesses that have a predominantly viral etiology, such as common colds and acute bronchitis [2-4].

Inappropriate use of antibiotics is of great public health concern, both nationally and globally, because of its association with increased antibiotic resistance in the community [5,6]. Bacterial strains that are increasingly resistant to antimicrobial agents pose a severe health threat, and of particular concern is community-acquired infections caused by multi-drug-resistant *Streptococcus pneumoniae *[7,8], macrolide-resistant *Streptococcus pyogenes *[9-11], and methicillin-resistant *Staphylococcus aureus *[12,13].

Antimicrobial resistance is an increasing problem worldwide, and several countries have implemented surveillance systems in recent years. The Norwegian surveillance program for antimicrobial resistance (NORM) was established in 1999 and has reported yearly on the epidemiology of antimicrobial resistance and the usage of antimicrobial agents [14]. Antimicrobial susceptibility data on common pathogens such as *Klebsiella *species, *Haemophilus influenzae*, *Staphylococcus aureus*, and *Streptococcus pyogenes *confirm that antimicrobial resistance is still a limited problem in Norway [15]. This also applies for the prevalence of macrolide-resistant *Streptococcus pneumoniae *(MRSP), which has increased dramatically in several European countries [16], but is still a relatively limited problem in Northern Europe [17,18].

Low levels of antimicrobial resistance in Scandinavian countries is probably due to relatively conservative antimicrobial prescription patterns, compared to southern Europe and USA [19]. Nevertheless, an increase in macrolide resistance in *Streptococcus pneumoniae *blood culture isolates has also been observed in Norway; from 2.4% in 2000, 4.8% in 2002 and 6.0% in 2003, to 9.7% in 2004 [14]. Macrolides are recommended as second-line alternatives in the treatment of RTIs, because the prevalence of non-penicillin-susceptible *Streptococcus pneumoniae *is below 3% in Norway [20]. They are recommended only for patients who are allergic to penicillin or are suspected of having atypical pneumonia or pertussis. Thus, the increase in MRSP is of clinical concern as well as public health interest.

In Norway, more than 90% of all antibiotic prescriptions are issued by general practitioners (GPs) [21], and 60% of these prescriptions are issued as treatment for common RTIs, for which the Norwegian national guidelines recommend penicillin V as the drug of choice [22]. Generally, the number of antibiotic prescriptions has raised with more than 30% in Norway during the last two decades [21].

Norwegian GPs' prescription patterns of antibiotics were studied (1988–89) in the Norwegian county of Møre and Romsdal [4,23], where more than 95% of the GPs in the county participated in the survey. Of all RTIs, acute bronchitis, otitis, and upper respiratory tract infection were the most frequently recorded diagnoses that prompted an antibiotic prescription. Penicillin V was the most commonly prescribed antibiotic for patients suffering otitis, upper respiratory tract infection, tonsillitis, and sinusitis, whereas erythromycin and tetracyclines were most frequently prescribed for lower RTIs [4]. Seven out of 10 children (aged 0–12 years) who were prescribed an antibiotic, received it as a result of a RTI [23]. More than eight out of 10 children who consulted a GP for sinusitis, tonsillitis, acute bronchitis, or pneumonia were prescribed an antibiotic [23]. For ear infections, an antibiotic prescription was given in 44% of all consultations, but this proportion increased to 64% when follow-up contacts for this diagnosis were excluded [23]. Penicillin V was the most frequently prescribed antibiotic for children with ear infection, upper respiratory tract infection, tonsillitis, sinusitis, and skin infections, whereas erythromycin was given to a considerable proportion of those with bronchitis or pneumonia [23].

Acute otitis media (AOM) is the most common bacterial infection in children [24]. In the Western world, most cases of AOM remit spontaneously and without complications [25]. The prevalence of antibiotic treatment for AOM varies in different countries, between 31% in The Netherlands and 98% in USA, Australia and New Zealand [26]. In The Netherlands, treatment of symptoms without antimicrobials has been adopted as the routine initial treatment for otitis media, and this policy is associated with decreased emergence of resistance among organisms commonly found in AOM [27]. In a meta-analysis, Del Mar et al. [28] concluded that early use of antibiotics provided only modest benefit for AOM, and that 17 children with AOM must be treated with antibiotics in order to prevent one child from experiencing pain 2–7 days after presentation of symptoms. It is likely that AOM is the reason for many unnecessary prescriptions for antibiotics, especially when broad-spectrum antibiotics are used [26]. In a study from a Norwegian emergency department setting, 90% of 819 children (aged 1–15 years) with AOM received treatment with antibiotics, and 18% of these prescriptions where for antibiotics other than penicillin V [29].

Sinusitis represents a complicated and multifaceted group of diseases that is generally classified into the categories of acute and chronic [30]. The diagnosis of acute sinusitis in GP settings is a challenging task [31], and although many instances of acute sinusitis are of bacterial origin, most cases are thought to be inflammatory rather than infectious [30]. The issues of the pathogenesis and treatment of sinusitis are ongoing areas of controversy and active investigation [30,31].

Steinman et al. [32] found that broad-spectrum antibiotics were prescribed in more than half of all cases of RTIs in adults, and that there was a wide variation in prescribing of such agents among different groups of patients and physicians, even after controlling for diagnosis and comorbidities. In a large, cross-national database study of antibiotic use and its associations with resistance, Goossens et al. [33] investigated outpatient antibiotic use in 26 countries in Europe that provided internationally comparable distribution or reimbursement data in the period 1997–2002, and found that prescription patterns varied greatly; the highest rate was in France and the lowest was in The Netherlands. A shift from the old narrow-spectrum antibiotics to the new broad-spectrum antibiotics (like the newer macrolides) were observed, as well as striking seasonal fluctuations with winter peaks in countries with high yearly use of antibiotics [33].

The use of macrolides for common airways infections deviate from current Norwegian national guidelines [34,35], and Brubakk and Bruun [36] underline that indications for macrolides in airway illnesses are limited to infections with *Bordetella pertussis *and *Mycoplasma pneumonia*.

In outpatients, macrolide consumption is closely linked to the development of streptococcal resistance. In Finland, there was a steady and statistically significant decline in erythromycin resistance among *group A streptococcal *isolates from throat-swab and pus samples after a reduction in the use of macrolide antibiotics in outpatient therapy [37]. Evidence from Sweden and New Zealand also demonstrate that a reduction in antibiotic use can be accompanied by a reduction in antibiotic resistance [38,39].

In a recent review issued by the Cochrane Collaboration, Arroll [40] argues that antibiotics have only a limited role in the treatment of AOM, sore throat, acute maxillary sinusitis, common cold, and acute purulent rhinitis. Except for radiologically proven acute maxillary sinusitis, antibiotics are not recommended as initial treatment for any of the conditions mentioned above [40].

Questions on validity and relevance of individual studies that are summarized in systematic reviews may be assessed differently by various reviewer groups, and may explain why the guidelines from the Infectious Disease Society of America (2002) recommend routine treatment for otitis media [41], whereas the Cochrane review represent a more cautious line and remain consistent with the older guidelines in not recommending antibiotics as a first-line treatment [40].

Over the past decade, general practice prescribing of antibiotics to children has halved in England, and this reduction has not been associated with an increase in admission to hospital for possible complications, like peritonsillar abscess or rheumatic fever [42]. The decline in use is due to a substantial reduction in prescriptions by GPs [42]. After 1997, the proportion of prescriptions taken to a pharmacist also declined, possibly indicating that GPs were adopting the "delayed prescribing" policy (issuing prescriptions with advice to parents to wait and see if their child's condition improved spontaneously) that was introduced after widespread dissemination of trial results supporting this practice [43].

An important aspect in the strategy of "delayed prescriptions" is the necessary management of patients' requests for specific treatments. Evidence suggests that a majority of patients feel that antibiotics are an appropriate treatment for a wide range of respiratory tract symptoms, and that doctors will prescribe antibiotics for them [44], despite convincing evidence of only limited antibiotic benefit for such conditions [25,40,45-53].

The first evidence of benefit from a randomized trial of delayed prescriptions for respiratory infections came from a trial of antibiotics for acute sore throat carried out by Little et al. [46], who gave either a prescription for antibiotics to be filled immediately, or one to be filled after three days, or no prescription for antibiotics. The "immediate group" consumed 99% of the antibiotic prescriptions, while the "delayed group" used only 31% with no apparent negative medical consequences [46]. In an other report, Little et al. [54] found evidence that both current and previous antibiotic prescribing for sore throat increased reattendance, and argues that doctors should avoid antibiotics or offer a delayed prescription for most patients with sore throat, in order to avoid medicalising a self-limiting illness.

Both Norwegian [55] and Danish [56] studies show great variation in GPs' prescription patterns for RTIs, and demonstrate a lack of adherence to current guidelines [21]. The potential for improvements are important, because community-wide antibiotic resistance reduces the effectiveness of currently available drugs to combat bacterial pathogens, and increases the individual patient's risk of becoming infected with drug-resistant organisms [57-59]. Previous studies have shown benefit from targeted educational intervention strategies towards GPs. Coenen et al. [60] reported that an (inter)actively delivered tailored intervention implementing a guideline for acute cough in a GP setting was successful for optimizing antibiotic prescribing without affecting patients' symptom resolution. Munck et al. [61] reported that a tailored audit of Danish GPs' antibiotic prescriptions for RTIs through the *Audit Project Odense (APO)*, resulted in a reduction in prescribed antibiotics, and that the large share proportion of broad-spectrum antibiotics was shifted to penicillin. The intervention effect in this study was significant, even two years after the intervention [61].

Welschen et al. [62] conducted a randomized controlled trial aimed at reducing antibiotic prescription rates for RTIs in primary care. The intervention included; a) group education meetings with consensus procedures and communication skills training, monitoring, and feedback on prescribing behaviour for GPs; b) group education for GPs' assistants and for pharmacists; and c) education materials for patients. At baseline, antibiotic prescription rates for RTIs were almost similar in the intervention and the control groups (27% and 29% respectively). Following the intervention, the prescribing rate in the intervention group fell to 23%, whereas it increased to 37% in the control group (mean difference of change; – 12%). The intervention did not influence patients' satisfaction or hospital referral rates [62].

Flottorp et al. [63] conducted a multifaceted intervention in 142 general practices to improve the management of urinary tract infections (in women), and sore throat. The intervention was based on patient educational material, computer based decision support and reminders, an increase in the fee for telephone consultations, and interactive courses for general practitioners and practice assistants. In this study patients in the sore throat group were 3% less likely to receive antibiotics after the intervention, compared to the control group [63].

In the Norwegian *Møre & Romsdal Prescription Study*, Rokstad et al. [64] performed a controlled educational intervention towards GPs, aimed at improving prescriptions for acute cystitis and insomnia. Their intervention included mailed prescription feedback along with printed therapeutic recommendations, and resulted in statistical significant (although clinically moderate) improvements in prescription patterns in the intervention group (GPs in one district), compared to the control group (GPs in another district) [64].

Eisenberg [65] has suggested that the core principle of effective feedback is individualized feedback with information on behaviour compared with specific standards: It seems as if such feedback is more effective when delivered face-to-face [65,66].

The results of evaluations of interventions to improve clinical practice vary and large effects are rarely seen [67,68]. Several educational strategies have been used. Davies et al. [69] reviewed 777 continuous medical education (CME) studies and found evidence that educational intervention consisting of passive dissemination of clinical practice guidelines had only little or no effects on practice. This corresponds with later reports by Oxman et al. [70] and Freemantle et al. [71]. Other, more active strategies, like educational outreach visits [72] and multifaceted interventions [67], are more effective, but will generally require more resources [67]. Soumerai et al. [73] reviewed educational methods for improving prescribing in primary care, and examined the approaches of feedback to prescribers, mailed-out educational material, group education and educational outreach (academic detailing), with educational outreach having the largest impact [66]. In an extensive review, Jamtvedt et al. [74] concluded that audit and feedback may be effective for improving professional medical practice, although the effects are generally small to moderate. Absolute effects of audit and feedback are more likely to be large when baseline adherence to recommended practice is low [74].

Underlying reasons for deviations between clinical practice and clinical guidelines vary from one problem to another and from one physician to another [70]. Therefore, it is recommended to address potential barriers to change when tailoring an intervention targeting change in medical performance. Because non-equivalent comparison groups may distort the results of evaluations, cluster-randomized trials (in which healthcare professionals or groups of professionals, rather than patients are randomized) are more likely to provide valid results than other research designs, such as controlled before-after or time-series studies [75].

### Objectives

The primary objective of this trial is to evaluate the effects of a tailored intervention towards Norwegian GPs for a more rational approach to antibiotic prescriptions for RTIs. Our hypothesis is that an educational intervention program carried out during a 6-month period will be effective in improving prescribing patterns by reducing the total number of antibiotic prescriptions, as well as reducing the relative amount of broad-spectrum antibiotics, with special emphasis on macrolides. Degree of improvement will be measured by prevalence of specific antibiotic prescriptions and the combination of particular antibiotic prescriptions and selected diagnostic codes, according to the International Classification of Primary Care (ICPC-2) [76]. Secondary objectives are to estimate the agreement between prescribed and actually dispensed drugs, the latter based on dispensed drugs recorded in the Norwegian Prescription Database (NorPD), a national registry including data for all prescription drugs issued at Norwegian pharmacies, established in 2004 [77]. We will also explore effect of "delayed prescriptions" on the antibiotic prescription patterns.

## Methods/Design

Our main hypothesis will be tested using a cluster-randomized controlled trial comparing outcomes between the intervention and control groups at follow-up. The clusters are existing peer CME groups, comprised by GP specialists. These groups of GPs will be randomized to receive a tailored intervention to support a more rational antibiotic prescribing for RTIs, or to a control group.

Prescription data will be collected for all eligible patients from intervention and control groups at baseline and one year after the initiation of the trial.

### Participants

In Norway, specialists in general practice must renew their clinical specialty every five years. In this renewal process, participation in a number of peer CME group meetings are compulsory, in order to stimulate a continuously medical education and reflection.

GP's in the same practice usually attend the same peer group, according to information from the Norwegian Medical Association. All peer groups (approximately 250) on average consisting of seven to eight colleagues located in the southern part of Norway, will be invited to participate in this trial. Our aim is to recruit approximately 80 peer groups or more to the current trial. Participating practitioners using one of four major electronic patient record (EPR) systems (Infodoc^®^, WinMed^®^, ProfDoc Vision^® ^or System X^®^) are eligible for the trial. More than nine out of ten of Norwegian general practices use one of these four EPRs, which are used routinely during consultations with patients [78].

### Intervention

We have developed an intervention through a process of identifying inappropriate prescriptions of antibiotics for RTIs; either as particular drugs, or drugs linked to specific diagnosis. Identification of irrational antibiotic treatment is based on guidelines on antibiotic prescriptions issued by The Norwegian Board of Health 2000 [35]. This guideline was distributed to all Norwegian GPs in 2000 and is also available on the Internet. Examples of suboptimal antibiotic prescribing according to this guideline are listed in Table 1.

In order to implement a more rational prescribing, 13 GPs will be recruited as tutors, each with responsibility for the intervention in three peer CME groups. Each tutor, named Prescription Peer Academic Detailer (Rx-PAD), will receive a four days' pre-study training program, focusing on the evidence of antibiotic treatment of RTI, pedagogical intervention techniques, and how to install and use the data-software program for data collection. When recruiting Rx-PADs, emphasis is also laid on the economic independence from any pharmaceutical manufacturers.

**Figure 1:**
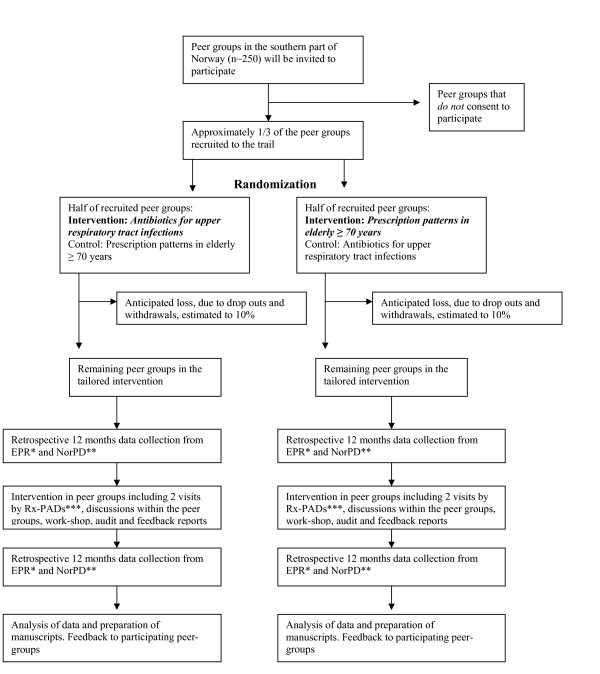
**Flow of practices through The Rx-PAD Study**. *Electronic Patient Record, **Norwegian Prescription Database, ***Prescription Peer Academic Detailer

The tailored intervention towards the GPs in the peer groups will include two educational outreach visits, both performed by the same Rx-PAD. The elements of the intervention are discussions within the peer group, collection of individual prescription data, audit based on individual feedback reports, as well as a one day regional work-shop, see Figure 1.

**Figure 2:**
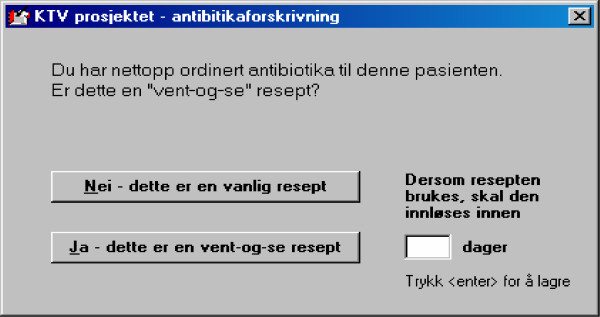
**Example of pop-up on physician's computer screen**. The pop-up appears when the physician is about to prescribe antibiotics for a respiratory tract infection (RTI), and enables the physician to decide whether the prescription is 1) to be dispensed immediately or 2) to be dispensed in a specified number of days, with advice to the patient to wait and see if the condition improves spontaneously, so-called "delayed prescription". Type of prescription and recommended delay (n days) for delayed prescriptions will be registered in the study software installed in the physicians computer and analysed by research staff.

In the first outreach visit, the main elements of the intervention will be presented, with special emphasis on evidence-based prescription of antibiotics for RTIs in out-patients, choice of first-line drugs, and treatment goals. During the visit, a software package will be delivered to each participant, for installation on own practice computer with EPR. This enables the participants to extract individual data from the preceding 12-month period. These data will be used for feedback reports and comprise the baseline data used in this study. For two of the four EPR systems (WinMed^® ^and ProfDoc Vision^®^), the software package will also include computerized "pop-ups" on the computer screen when antibiotics are prescribed, in order to mark prescriptions as either "regular" (to be dispensed immediately) or "delayed" (to be dispensed in n days) (Figure 2). Data from these "pop-ups" will be integrated in the second data extraction, and will represent a separate factor in the subsequent data analyses. These two EPR systems are probably used by more than half of all GPs involved in this study. Prescription data will be collected on diskettes and analyzed by research staff, followed by an individual prescription report, which will be mailed to each participant.

The second outreach visit, which will take place within two months after the first visit, focus on the newly revealed prescription patterns. Rx-PADs will facilitate the discussion within the peer groups, based on individual feedback reports, enabling participants to compare own prescription patterns with overall averages. This will trigger discussion within the peer group, aimed at critical reflection towards own prescription strategies for RTIs and the disclosure of areas where individual improvements are desirable and possible.

About three months after the second outreach visit, all participants will be gathered in regional work-shops where evidence-based rationale behind pharmacological treatment of RTIs in out-patients will be outlined in more depth on the basis of baseline prescription data. Twelve months after the first data extraction, a second data extraction of the GPs' prescribings for the preceding 12-months period will be undertaken and new individual reports will be sent to the participants.

### Data handling

Software aimed at extracting predefined data sets will be developed for this trial. The software will be compatible with the four EPRs (Infodoc^®^, WinMed^®^, ProfDoc Vision^® ^or System X^®^) used by the large majority of Norwegian GPs. The dataset from each individual GP will provide information on number of patient appointments, and main diagnosis linked to each appointment with patients with a respiratory tract infection. Diagnosis will be based on the International Classification of Primary Care (ICPC-2) [76]. Extracted data will also contain drug prescription details, described in terms of The Anatomical, Therapeutic, Chemical (ATC) classification system with Defined Daily Doses (DDDs), in short: The ATC/DDD system [79].

In order to merge prescription data provided by NorPD with data from the EPR systems, the Civil Personal Registration (CPR) number for each patient will be extracted from the EPR. These are the unique identification numbers for Norwegian citizens, and will be deleted from the research database after the record linkage has been performed. The dataset from NorPD will provide data on drugs actually dispensed.

**Figure 3:**
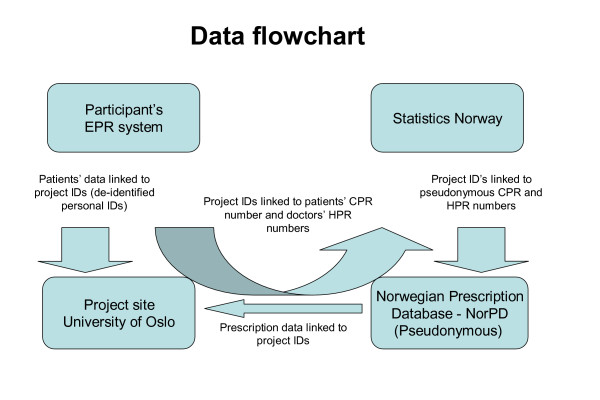
**Logistics of data collection**. Flow-chart of merging process of prescription data provided by the Norwegian Prescription Database (NorPD) and data from the electronic patient record (EPR) systems. Patients' Civil Personal Registration (CPR) numbers are unique Norwegian residents' identification keys. Health Personnel Registration (HPR) numbers are unique Norwegian health personnel registration keys. Identifiable data will be deleted from the research database when the merge is completed, as the de-identified personal IDs will be sufficient for the subsequent data analysis. Statistics Norway, which is the public institution in Norway responsible for collecting, analyzing and disseminating official statistics, will provide CPR and HPR pseudonyms making it possible to merge the two data sources.

Extracted data from each EPR will be encrypted, stored on a diskette and mailed to the principal researcher. A flow-chart of the data collection is summarized in Figure 3.

### Outcome measures

Primary outcome measures will be changes in prescription patterns after the tailored educational intervention in terms of prescriptions for antibiotics in out-patients with RTIs. The intervention will include the option of "delayed prescriptions". Following the intervention, prescription data will be collected one year after the first (baseline) gathering of prescription data. Change in prescription patterns in the intervention group will be compared with the non-intervention control group, and with a sample of Norwegian GPs not included in the current trial. The latter data will be provided by the NorPD. Outcome measures are summarized in Table 2.

### Pilot study

The prescription data extraction procedures and the two peer group visits will be piloted in one peer CME group, before the main trial.

### Recruitment and randomization

Block-randomization of peer CME groups will be done within geographical strata, each comprised of one or more counties. The size of the blocks will vary according to the number of recruited groups within each stratum. An independent researcher not involved in the study will be responsible for the randomization. Based on this randomization, peer groups will be assigned to either the intervention group or to the control group.

### Ethics and data security

Physicians participating in the peer CME groups will be given information about the objectives of the study and the practical impact it may have on their practice. They will be told that each peer CME group will be randomized to one out of two interventions (improved antibiotic prescriptions for RTIs or improved prescription patterns in elderly ≥ 70 years), and that they will serve as control group for the intervention they do not take part in. Participation is based on written informed consent from all physicians. The project has been presented for The Regional Committee for Research Ethics and approval from The Norwegian Social Science Data Services (NSD) has been obtained, implicating acceptance to extract prescription data. In order to use patient identification data in the merging process between NorPD and EPR databases, The Directorate for Health and Social Affairs has approved dispensation from the health-professional secrecy.

### Sample size and statistics

A prevalence of 27% of inappropriate prescriptions of antibiotics for RTIs in general practice settings were found in two previous studies; one from Norway [80] and one from The Netherlands [62]. In accordance with these findings [62,80], we anticipate a baseline prevalence of inappropriate prescription of antibiotics for RTIs of 27% in the present study.

Flottorp et al. [63] demonstrated a 3% reduction of inappropriate antibiotic prescription for sore throat in a tailored intervention in general practice settings. This intervention was not, however, based on peer CME groups. This was considerably less than the 12% reduction (absolute value) of antibiotic prescription rates for RTIs found by Welschen et al. [62], in a peer group intervention trial.

In the present study we will consider a reduction of inappropriate prescriptions for RTIs by one third to be clinically significant. Assuming that the prevalence of inappropriate prescriptions in the control group remains constant, this equals a reduction from a baseline level of 27% to a post-intervention level of 18% or less (9% absolute reduction; 33% relative reduction).

The sample size calculation needs to take into account and adjust for intra-cluster (peer group) correlation, since the data within peer groups cannot be considered independent. The intra-cluster correlation is assumed to be 0.085, based on data from previous studies [63,78]. Furthermore, we expect an average of 7 participating doctors per peer group (allowing for some withdrawal) and an average of 300 cases of RTIs per GP during the intervention period, amounting to a total of 2100 cases per cluster. The variance inflation factor [81], used to correct the sample size, was therefore 179.

Based on these figures and applying an 80% power level and a 5% statistical significance level, we estimate an intervention sample size requirement of 31 peer CME groups and a corresponding number of control groups.

We will carry out post-test comparisons between the intervention and control group using cluster-adjusted chi-square and *t*-tests. Analysis of covariance may be used to adjust for imbalance of baseline levels between the intervention- and control-groups [82]. The data set will primarily be analysed according to the cluster-randomization, however, analyses at physician- and sub-groups of physicians will also be performed, based on individual patients' data.

## Discussion

A thorough evaluation of the diagnostic indications for antibiotic treatment of RTIs may impose important benefits, whereas inappropriate prescribing may lead to undesirable consequences like the development of antibiotic resistance. We have therefore developed an intervention aimed at supporting the implementation of more rational antibiotic prescribing patterns for RTIs in GP settings and planned a trial to assess the effectiveness of this intervention.

The control group in this study will not receive any intervention towards prescriptions of antibiotics, but will be assigned to another drug prescription trial, where they will recieve an educational intervention for improving their prescribing (other than antibiotics) for elderly outpatients ≥ 70 years [83]. As such, the control group may be susceptible to changes in their general prescription patterns, i.e. a possible Hawthorne effect. To control for this, we will be able to analyze prescription patterns for *all *Norwegian GPs not included in the current trial, using data from the NorPD [77]. These data, however, will not be linked to diagnoses, and the comparison will therefore be performed on total prescription of the different antibiotics, except those exclusively used for urinary tract infections.

To assure a highest possible response rate from the participating GPs, we will provide technical telephone support during the data collection period. Furthermore, the participants must provide data to receive an individual feedback report. Also, the Rx-PAD will remind their group participants to send in data sets before the second peer group meeting.

Because the aim of this study is to improve quality according to good clinical practice, the study is not likely to imply risks or impaired quality of care for the patients involved. The risk of data-misuse is limited due the fact that the research database will only be linked to de-identified patient ID numbers.

The estimated ICC-value, which will be used in the statistical analyses, reflects the three different levels of clustering; each participating doctor, each clinic, and each peer CME group. Our ability to establish an exact ICC-value is limited, due to few comparable studies.

A reduction of inappropriate prescriptions by one third compared to the baseline figures may seem too optimistic. However, in the light of a previous study [62], we still believe that this is a realistic goal.

## Competing interests

SG has interests in Mediata Ltd, the company developing software for the project. The other authors declare that they have no competing interests.

## Authors' contributions

ML and JS had the original idea for the study. SG, ML and JS prepared the initial draft of the study protocol. All authors have participated in the planning of the study. AF prepared the manuscript in cooperation with all authors, who have all approved the final version of this manuscript.

## Pre-publication history

The pre-publication history for this paper can be accessed here:



## Figures and Tables

**Table 1:** Examples of inappropriate antibiotic prescription patterns according to guidelines by The Norwegian Board of Health 2000

Uncomplicated acute otitis media (AOM) in children > 1 year of age with symptoms < 3 days
Acute sinusitis with moderate symptoms < 7 days
Acute tonsillitis without positive indication of infection caused by *group A Streptococcus*
Acute bronchitis (except verified infections caused by *Mycoplasma pneumonia *or *Chlamydia pneumonia*)
Use of broad spectrum antibiotics where penicillin V could be prescribed

**Table 2:** Outcome measures in study of the effect of a pedagogic intervention towards groups of Norwegian GPs, concerning antibiotic prescription for respiratory tract infections (RTIs)

Baseline data collected during one year:
Proportion of consultations with antibiotic prescription of all consultations with respiratory tract diagnoses
Proportion of consultations with antibiotic prescription of consultations with specified RTIs
Proportion of consultations with narrow-spectrum antibiotic prescription of all antibiotic prescription
Predictors for high and low antibiotic prescription
Change as measured one year after pedagogic intervention:
Change in total antibiotic prescription for respiratory tract diagnoses
Change in proportion of consultations with antibiotic prescription of all consultations with respiratory tract diagnoses
Change in proportion of consultations with antibiotic prescription of consultations specified RTIs
Change in proportion of consultations with narrow-spectrum antibiotic prescription of all antibiotic prescription
Predictors for change in total antibiotic prescription and proportion narrow-spectrum antibiotics

Use of delayed prescriptions:
Proportion of use of delayed prescriptions by all antibiotic prescriptions for respiratory tract diagnoses
Proportion of use of delayed prescriptions by antibiotic prescriptions for specified respiratory tract diagnoses
Predictors for high/low use of delayed prescriptions
Comparisons between antibiotic prescriptions and data from the Norwegian Prescription Database (NorPD)
Proportion and delay of dispensed antibiotic prescriptions compared to prescribed antibiotics

Refund of antibiotics:
Proportion of antibiotics prescribed with full refund
